# Integrating skin and vasculature in a Multi-Organ-Chip Platform

**DOI:** 10.1186/1753-6561-9-S9-P20

**Published:** 2015-12-14

**Authors:** Katharina Schimek, Annina Markhoff, Frank Sonntag, Martin Blechert, Roland Lauster, Uwe Marx, Gerd Lindner

**Affiliations:** 1 TU Berlin, Institute for Biotechnology, Faculty of Process Science and Engineering, 13355 Berlin, Germany; 2 Fraunhofer IWS Dresden, 01277 Dresden, Germany; 3 Fraunhofer IZM Dresden, 13355 Berlin, Germany; 4 TissUse GmbH, 15528 Spreenhagen, Germany

## Background

Tests for drug development require an almost perfect fit with the human (patho-) physiological microenvironment. The majority of skin equivalents currently commercially available are based on static culture systems emulating only human epidermis, or combining epidermis and dermis in so-called full thickness skin equivalents. None of the existing systems contain important elements, such as vasculature, skin appendices or an immune system. Therefore, current in vitro and animal tests are failing to accurately predict drug toxicity. Our Multi-Organ-Chip (MOC) platform is a micro scale bioreactor providing pulsatile dynamic perfusion for microscale organoids. Here, we combine skin equivalents with vasculature in our two-organ variant (2OC). This would be needed for physiological-like interactions, regulation and, eventually, homeostasis within the chip.

## Material and methods

Skin equivalents were build up using 7mm punch biopsies of Matriderm® (Asclepios Medizintechnik). Human dermal fibroblasts (6*105/cm2) were pipetted onto the top of the scaffolds and cultivated in 5% CO2 at 37°C for 7 days. Keratinocytes (5*105/cm2) were then added as a drop onto the matrix. After 1h incubation for attachment of the cells, skin equivalents were cultivated submerged for another 7 days. Then, skin equivalents were lifted to the air-liquid interface and cultivated for further 14 days. After 28 days of static culture, the skin equivalents were added to the MOC (day zero of MOC culture).

**Figure 1:**
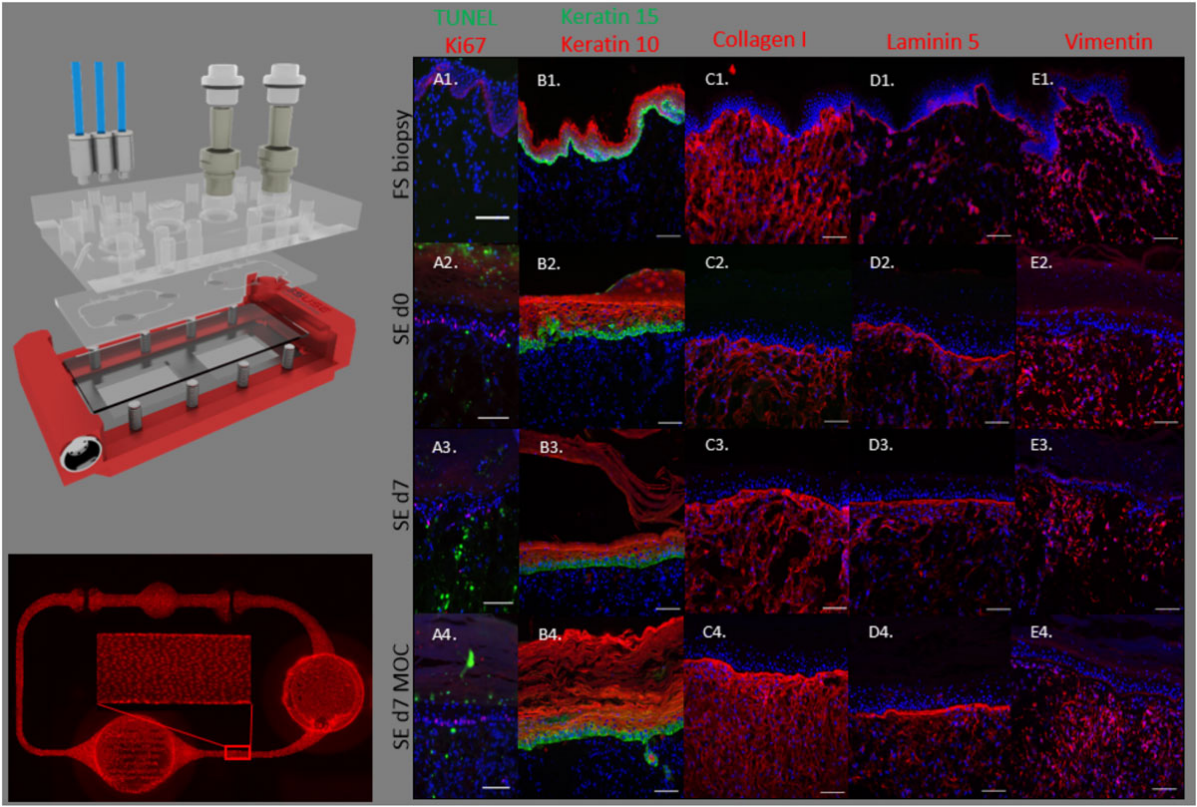
**Skin and vasculature co-culture in the MOC**. a) Exploded view of the MOC platform comprising a polycarbonate adapter-plate and a PDMS chip accommodating two microfluidic circuits (footprint: 76 mm × 25 mm; height: 3 mm) each with one culture compartment and one Medium reservoir. **b) **Microvascular circuit in the MOC after 7d co-culture. Functionality of the microvascular vessel system demonstrated by live cell viability staining (CellTrace™Calcein red-orange AM assay; red). **c) **Characterization of skin tissues. A1-E1 native foreskin (FS) biopsy. A2-E2 skin equivalent (SE) at d0 of MOC culture. A3-E3 SE cultured statically outside the MOC for 7 days. A4-E4 SE cultured in the MOC platform for 7 days. TUNEL/Ki67 staining showing apoptotic and proliferating cells, respectively (A). Functionality of SE shown by K10/K15 (B), Collagen I (C), Laminin 5 (D), and Vimentin (E) expression.

The MOC platform (Figure 1a) provides a constant pulsatile flow of medium via its built-in micropump and ensures oxygen and nutrient supply. The system is filled with up to 600 µL of medium. No external reservoirs need to be attached that would otherwise dilute the enriched medium.

Human dermal microvascular endothelial cells (HDMECs), isolated from human foreskin, were injected into the microfluidic channel system. After even cell distribution inside the circuit, the device was cultivated at 37°C under static conditions for 3 h to allow the cells to attach to the channel walls. A frequency of 2 Hz was applied for continuous dynamic operation and, after 3 days of monoculture, the skin equivalent was added for co-cultivation for another 7 days.

## Results

Functionality of the microvascular vessel system was demonstrated by live cell viability staining (Figure 1b). Endothelial cells maintained a confluent monolayer after 7 days of dynamic co-culture. HDMEC in the Channels were elongated and oriented into the direction of flow.

In comparison to cultures utilizing conventional static conditions, skin equivalents cultivated in our perfused MOC system together with the endothelial cells showed improved vitality. There were more viable (TUNEL-positive) and proliferating (Ki67-positive) cells in the SEs cultured in the MOC compared to the SEs cultured under static culture conditions. Expression of specific basal membrane and extracellular matrix proteins (Collagen IV, Laminin 5, Collagen I) and defined mesenchymal and epithelial markers (Vimentin, CK10/15) showed remarkable consistency after 7d of MOC co-culture (Figure 1c).

## Conclusions

These results render the MOC used a useful tool for long-term co-culture of skin equivalents and endothelial cells, keeping most of their structures undamaged

The on-chip micro-pump was able to provide a suitable shear stress environment for the endothelial cells. Dynamic perfusion of the MOC mediated the elongation with the direction of flow. HDMECs completely covered the channel walls of the entire circuit and remained viable after 7d of co-culture. Additionally, skin equivalents cultivated in our perfused MOC system together with the endothelial cells showed improved vitality and consistency of most of their structures.

In vitro testing of substances using the MOC, whether applied topically or into the medium, might be performed with significantly prolonged test periods, enhanced validity and online endpoint analysis compared to static cultures. Ultimately, combining different approaches, such as vascularization and integration of the MF model into skin equivalents, will provide the most predictive in vitro model of skin with vasculature and hair follicles so far.

## Acknowledgements

This project is funded by the German Research Foundation (DFG), LA 1028/7-1

